# Parents’ attitudes towards and perceptions of involving minors in medical research from the Japanese perspective

**DOI:** 10.1186/s12910-018-0330-1

**Published:** 2018-11-21

**Authors:** Yasue Fukuda, Koji Fukuda

**Affiliations:** 1 General Medical Education and Research Center, Teikyo University, 2-11-1 Kaga, Itabashi-ku, Tokyo, 174-8605 Japan; 2 School of Political Science and Economics, Waseda University, 1-6-1 Nishi Waseda Shinjyuku-ku, Tokyo, 169-8050 Japan

**Keywords:** Children, Minors, Medical treatment, Medical research, Informed consent, Informed assent, Parental attitude, Parental permission

## Abstract

**Background:**

Children’s intentions should be respected. Parents are the key persons involved in decision-making related to their children. In Japan, the appropriate ages and standards for a child’s consent and assent, approval, and decision-making are not clearly defined, which makes the process of obtaining consent and assent for clinical research complex. The purpose of this paper is as follows: to understand the attitudes and motives of parents concerning children’s participation in medical research and the factors influencing their decision-making. We also sought to clarify who has the right to be involved in decisions regarding children’s participation in research.

**Methods:**

A semi-structured Internet survey on parents’ opinions and attitudes and preferences concerning medical research involvement was conducted. Children were divided into three age groups (6–10-year-olds, 11–14-year-olds, and 15–18-year-olds), with three illness severity categories. Possible correlations between the number of children, children’s ages, parents’ educational levels, and parents’ attitudes were examined.

**Results:**

Among the participants, 42.3% recognized the term “informed consent.” The proportion of participants who understood “informed consent” increased with educational level. Four out of five participants did not know, or had not heard of, the term “informed assent.” Furthermore, the percentage of those who understood the term “informed assent” increased with academic level. Participants generally believed in prioritizing parents’ opinions over children’s, and that parents and children would ideally reach a joint decision. Although many parents favored collaborative decision-making, they also wanted their own will reflected in the decision and felt they should receive important information before their children do. Decision-making was affected by the condition’s severity and prognosis. This indicates that most Japanese parents believe that their children have the right to know their disease name and treatment; nonetheless, they should be protected. Parents’ values and judgments regarding medical intervention involving their children varied.

**Conclusions:**

Children’s ability to consent to treatment and research believed to be in their best interests should be assessed appropriately. They should be permitted to provide consent or assent, and their views should be respected. Involving children in decision-making fosters more open communication and transparency between medical professionals, parents, and children.

**Electronic supplementary material:**

The online version of this article (10.1186/s12910-018-0330-1) contains supplementary material, which is available to authorized users.

## Background

The importance of respecting the will of children and young people has been noted [1–4]; however, the process of children’s informed consent and assent has not been fully clarified [5, 6]. “Informed consent” refers to the legal approval for medical intervention provided by a competent child, whereas “informed assent” implies agreement to intervention provided by a child who does not have the full capacity to consent, and it therefore does not constitute legal approval [7–10].

Informed assent participants are those who are objectively judged as having insufficient judgment ability to give informed consent. In cases of informed assent, children receive explanations of the research that are appropriate for their level, and they give approval based on their degree of understanding. However, because informed assent given by children does not have binding legal force, parental permission is required in such cases [11].

In order to respect children as individuals, they themselves need to be able to select and participate in research and treatment methods. On the other hand, due to the children’s lack of social experiences and cognitive ability, it is not always possible for them to understand the medical personnel and researchers’ explanations sufficiently [12].

As a result, it is possible that they cannot know what is in their best interests. This raises the question of who will evaluate children’s abilities to agree to what is in their best interests.

The development of pediatric medicine has long been neglected and is known as a “therapeutic orphan” [13, 14]. This is because there is a diverse range of subjects covering the newborn to adolescent periods, and detailed responses, involving such things as dosage forms and pharmacokinetics, are required for each age group. Special consideration is necessary for the planning of clinical trials and the acquisition of consent in pediatric research and trials [15]. Thus, there are ethical and practical issues that need to be addressed when assessing parents’ and children’s intentions.

Rather than excluding minors from clinical trials, it has become necessary to compensate for the lack of research into pediatric treatment methods by promoting minors’ participation in clinical trials [16, 17]. In 2007, the development of pediatric medicine was enhanced by a childhood medicine regulation by the European Union [18].

In 2002, the Best Pharmaceutical for Children Act (BPCA) was enacted in the United States with the aim of encouraging and securing the safety of pediatric medicine, and it gave pharmaceutical companies an incentive (pediatric exclusivity) to extend the period of patent protection to 6 months [19]. In Japan, a much-needed conference was held in 2010 to review unconfirmed drugs and non-exempt drugs. A clinical trial network was launched in the same year to promote clinical trials and improve pediatric medicine [20]; however, there are still no regulations that legally oblige pediatric drug development. Consequently, the inappropriateness of the medication formulation for use with children is regarded as a problem.

Therefore, we studied which of the following decision-making models is preferred by parents and more applicable to the situation under question: one wherein parents make the final decision, a consensus model wherein the parent and child jointly agree, or an autonomous respect model that values the child’s autonomy.

A distinction needs to be made between studies with direct benefits and those without such benefits. Therapeutic research, for example, may have direct benefits for patients’ treatment and prevention, while nontherapeutic research may aim to support *future* treatment and prevention. Indeed, clinical trials can have treatment benefits for patients. It should be noted, however, that there is no need for research if a known direct benefit exists [21]. Moreover, there can be a degree of uncertainty about the existence of potential benefits for the patient. Therefore, we defined “medical research” in our study and presented the meaning of *“*medical research” to the participants as follows: “The medical research is not for the purpose of treatment or prevention for patients themselves but to contribute to the development of future prophylactic and therapeutic methods.”

Confirming parents’ intentions and inclinations is essential to building positive and trusting relations between medical professionals and patients as well as their families [22–24]. In order to examine parents’ backgrounds, intentions, and inclinations, which highly affect children’s decisions, an attitude survey was conducted with the parents of minors. This study was designed to investigate parents’ opinions and attitudes regarding their children’s assent or consent to participate in pediatric research.

## Methods

A semi-structured questionnaire was designed and implemented to survey the opinions and attitudes of parents of minors regarding treatments and medical research involving their children, as well as the question of who should provide consent.

Informed consent and assent both involve 1) explaining the medical procedure in a manner appropriate to the subject’s level of comprehension, 2) securing understanding from the subject, and 3) decision-making involving the subject, his or her guardian, or the subject and parents or guardians together [25].

The questionnaire focused on the explanation provided to the child, the child’s comprehension, and the provision of consent. If the respondents had several children, the age of the child to whom the respondent referred would be unknown; therefore, the questionnaire asked the respondent to mention the age of the child. The children to whom the respondents referred were divided into three age groups (6–10-year-olds, 11–14-year-olds, and 15–18-year-olds), with three categories of illness severity levels, whether or not total recovery was possible, the possibility of disability, and intentions to participate in a research study being set. Pearson’s chi-squared test was performed to examine the relationships of these factors with the person who makes the final decision.

### Recruitment of participants

The recruitment for the questionnaire received partial support from Cross Marketing Inc., an Internet survey company that received the privacy certification given to companies that comply with Japan’s personal information protection law. An online questionnaire was administered to parents of children aged 6 to 18 and are registered in the database. We recruited the parents (respondents) using a registered online survey panel from Cross Marketing Inc. The recruitment of the respondents was randomized from among the total number of respondents based on background information. We set the target number to 1200. In light of registered background information, such as age, gender, and academic background, answers that were false or incorrect were excluded from the data. In order to correct bias due to this being an Internet survey, stratified sampling was applied based on household income distribution to ensure the sample was similar to the average nationwide distribution in Japan as reported in the 2015 Comprehensive Survey of Living Conditions [26]. The Internet survey content was verified from February 10 to 19, 2017. Responses were collected online from February 24 to 28, 2017.

We fully analyzed completed questionnaires submitted by parents who gave their consent to participate in the survey.

We investigated participants’ demographic characteristics (i.e., age, gender, family composition, number and age of children, household income, and employment). Participants who gave inconsistent responses regarding family composition and the number of children were excluded; 1150 valid responses were retained.

### Statistical analysis

All data were summarized using interquartile ranges for continuous variables and counts and percentages for the categorical variables. Descriptive statistics were computed to examine the information provided to the children and parents’ attitudes towards them participating in research studies.

Parents’ gender and attitudes were compared using a Mann-Whitney U test. Pearson’s chi-squared test was used to evaluate the relationships between the number of children, the children’s ages, parents’ educational levels, and the parents’ attitudes.

All statistical inferences were made two-sided at a 5% significance level. All statistical analyses were performed using SPSS ver. 24. (SPSS Inc., Chicago, IL, USA).

## Results

### Participants

Parents’ demographic characteristics are shown in Table 1. There were 672 fathers (58.4%; mean age: 49.3 years) and 478 mothers (41.6%; mean age: 40.8 years).

**Table 1:** Demographic characteristics of parents and their child

		Number	(%)
Parent’s relationship with child			
Father		672	58.4%
Mother		478	41.6%
Parent’s age (mean, S.D)		42.6 (7.1)	
Father		43.9 (7.1)	
Mother		40.8 (6.7)	
Age of first child (mean, S.D)		12.2 (3.9)	
Age of second child (mean, S.D)		8.1 (4.3)	
Number of children			
1		448	39.0%
2		524	45.6%
≥ 3		178	15.5%
History of being admitted to hospital	Exists (Yes)	227	19.7%
	Non-existent	923	80.3%
Children’s experience being admitted to hospital	Exists (Yes)	206	17.9%
	Non-existent (No)	944	82.1%
Academic history (education level)			
Middle school		21	1.8%
High school		298	25.9%
Professional school		170	14.8%
University		531	46.2%
Graduate school		60	5.2%
Others		70	6.0%

### Awareness of informed consent, informed assent, and parents’ educational levels

Table 2 shows the relationship between the awareness of informed consent and parents’ educational levels.

**Table 2:** Degree of recognition of terms “informed consent/assent” and parents’ educational level

Educational level	Knows what it is Number (%)	Have heard about it but does not know what it entails Number (%)	Does not know what it is Number (%)
Middle school	3 (14.3%)	5 (23.8%)	13 (61.9%)
High school	75 (25.2%)	65 (21.8%)	158 (53.0%)
Professional school	61 (35.9%)	33 (19.4%)	76 (44.7%)
University	282 (53.1%)	118 (22.2%)	131 (24.7%)
Graduate school	41 (68.3%)	10 (16.7%)	9 (15.0%)
Others	19 (32.8%)	13 (22.4%)	26 (44.8%)
Does not want to answer	5 (41.7%)	3 (25.0%)	4 (33.3%)

Up to 42.3% (*n* = 486) of the participants knew the term “informed consent.” However, some respondents stated that they had heard of the term but did not understand what it was (21.5%), or they had not heard of the term or did not understand what it meant (36.4%), indicating that more than half the respondents did not understand what the term entailed. The proportion of participants who understood the term “informed consent” increased as educational level increased.

On the other hand, the proportion of respondents who had awareness of “informed assent” was merely 18.0%. Of these, 23.0% had heard of the term but did not know the details of it, which means that 60% of the respondents—four in five participants—either did not know the word, or they had heard of it but did not understand the concept. The breakdown of awareness of “informed assent” by academic level was as follows: junior high school (0%), high school (10.1%), professional school (15.9%), university (30.0%), and graduate school (30.0%), showing that the percentage of subjects who understood the term “informed assent” increased with academic level (*P* < 0.001) (Table 3).

**Table 3:** Recognition level of the terms “informed assent” and parents’ educational level

Educational level	Knows what it is Number (%)	Have heard about it but does not know what it entails Number (%)	Does not know what it is Number (%)
Middle school	0 (0%)	5 (23.8%)	16 (76.2%)
High school	30 (10.1%)	72 (24.2%)	196 (65.8%)
Professional school	27 (15.9%)	34 (20.0%)	109 (64.0%)
University	126 (30.0%)	119 (22.4%)	286 (53.9%)
Graduate school	18 (30.0%)	10 (16.7%)	32 (53.3%)
Others	5 (8.6%)	9 (15.5%)	44 (75.9%)
Does not want to answer	1 (8.3%)	4 (33.3%)	7 (58.3%)

### Attitudes towards providing an explanation to the child

We used a 5-point Likert scale with the options “agree,” “somewhat agree,” “neutral,” “somewhat disagree,” and “disagree.” Regarding the statement, “treatment method and research participation should be explained to the children,” 2.0% (*n* = 23), 6.6% (*n* = 76), 39.7% (*n* = 457), and 20.9% (*n* = 240) selected “disagree,” “somewhat disagree,” “somewhat agree,” and “agree,” respectively, with a mean Likert score of 3.7 ± 0.9, indicating that over 60% of the parents believed such information should be explained to the children.

With respect to gender, there were significant differences regarding how children should be informed: 56.7% of the fathers and 66.1% of the mothers responded “agree” or “somewhat agree,” respectively, with the statement “information should be given,” with more mothers answering that “the child should be informed” (Mann-Whitney’s U test: *p* < 0.05).

Concerning the question, “whether or not the explanation should be given to the children first before the parents,” 12.6% (*n* = 145) and 20.4% (*n* = 235) disagreed and somewhat disagreed, respectively that is, a total of 33% disagreed. In contrast, 18.1% (*n* = 208) somewhat agreed and 7.3% (*n* = 83) agreed; the mean Likert score was 2.97 ± 1.08, with a total of 25.4% agreeing. Although there were individual differences, the findings suggest a tendency for respondents to feel that medical procedures should be explained to parents first, indicating the necessity for parental involvement (*p* < 0.001).

The statement “it is better not to explain the severity of the disease” attracted divided opinions: 6.3 and 21.2% responded that they “agree” and “somewhat agree,” respectively, whereas 3.7 and 9.7% responded that they “disagree” and “somewhat disagree,” respectively.

Regarding the statement “the child should be informed of the risks of the treatment and research participation,” 11.1 and 33.7% responded that they “agree” and “somewhat agree,” respectively, whereas 1.2 and 7.4% responded that they “disagree” and “somewhat disagree,” respectively. The percentage of mothers who answered that they needed explanations of the risks of the treatment and research (14.4 and 32.6% selected “agree” and “somewhat agree,” respectively) was significantly higher (*p* < 0.01) than that of fathers (8.8 and 34.4% chose “agree” and “somewhat agree,” respectively).

Most agreed that an explanation should be given to children according to their comprehension ability with 35.5 and 41.7% responding “agree” and “somewhat agree,” respectively), and only 1.0% choosing “disagree” and 1.3% opting for “somewhat disagree.” The percentage of mothers who answered that they needed explanations according to their comprehension ability (44.4 and 40.0% “agree” and “somewhat agree,” respectively) was significantly higher (*P* < 0.01) than that of fathers (28.9 and 42.9% “agree” or “somewhat agree,” respectively).

Regarding the statement “information guidelines are necessary,” 27.1 and 59.3% responded “agree” and “somewhat agree,” respectively, indicating that most participants regarded some type of policy or guidelines as necessary.

These results suggest that parents think information should be adjusted to suit the patient, although opinions were divided regarding whether disease severity should be explained. Participants also generally considered it preferable not to inform children before their parents.

### Attitudes towards research participation and motivation to participate in pediatric research

Responses were divided regarding the statement “if there are no other methods of treatment, it is better to enroll the child in research”; 6.5 and 20.4% responded “agree” and “somewhat agree,” respectively, whereas 4.3 and 9.4% responded “disagree” and “somewhat disagree,” respectively. This indicates that more parents are willing to give consent to research for reasons other than their own child’s health than to refuse to do so.

Regarding the reasons why parents consented to research, in response to “For advanced pediatric research and future children treatment,” 11.0 and 39.4% responded with “agree” and “somewhat agree,” respectively, whereas 43.0, 3.8, and 2.8% responded with “neither agreed nor disagree,” “somewhat disagree,” and “disagree,” respectively. Further, 14.6% agreed with the statement “I hope that participation in research will lead to the treatment of my child,” while 37.3, 42.4, 3.0, and 2.6% stated “somewhat agree,” “neither agree nor disagree,” “somewhat disagree,” and “disagree,” respectively.

### Attitudes towards decision-making model (decision-making model preference)

Table 4 shows the relationship between the consent age of the children and their parents’ views regarding decision-making in research on children.

**Table 4:** The main person who decides on research study participation and decision style

consent age of the child (years)	The child Decides Number (%)	Consensus building Number (%)	The parents decide Number (%)	Unknown Number (%)
Non-life-threatening disease/illness that can be completely cured				
6–10	18 (9.0%)	85 (42.3%)	86 (42.8%)	12 (6.0%)
11–14	59 (14.1%)	204 (49.6%)	113 (27.5%)	35 (8.5%)
15–18	103 (19.1%)	245 (45.5%)	117 (21.7%)	73 (13.6%)
Non-life-threatening disease/illness but will leave a disability				
6–10	5 (2.5%)	90 (44.8%)	93 (46.3%)	13 (6.5%)
11–14	25 (6.1%)	217 (52.8%)	135 (32.8%)	34 (8.3%)
15–18	56 (10.4%)	288 (53.6%)	122 (22.7%)	72 (13.4%)
Life-threatening serious disease/illness				
6–10	4 (2.0%)	58 (28.9%)	123 (61.3%)	16 (8.0%)
11–14	9 (2.2%)	194 (44.8%)	172 (41.9%)	46 (11.2%)
15–18	33 (6.1%)	246 (45.7%)	182 (33.8%)	77 (14.3%)

A weak positive correlation was found between consent age and decision-making (*R* = 0.092; *p* = 0.0001). The higher the age group, the more likely parents were to choose the consensus-building approach or allow the child full autonomy.

Although many parents favored collaborative decision-making between parents and children, they also wanted parents’ will to be reflected in the decision.

Parents’ values and judgments regarding medical activities involving their children were varied. Decision-making was affected by whether the condition was life-threatening, whether it could be fully treated, whether it may result in disability; these results indicate that most Japanese parents believe their children have the right to be informed about their disease and medical research; nonetheless, they should be subject to protection.

Respondents who believed the child should decide made the following comments: “children should decide on things concerning themselves,” “we should hear their opinion since they should be respected as an individual,” “life consists of choices made by oneself; children are not the possessions of their parents,” and “children also have rights.” In contrast, parents who considered that the right to decide lays with the parents stated the following: “I don’t want my child to carry this responsibility,” and “children do not have sufficient comprehension of the situation.”

## Discussion

This study found that the degree of awareness of informed consent was approximately 40%, and awareness of assent was much lower, at 18%, but parents with higher educational levels had a higher awareness of both informed consent and assent.

However, no correlations were found between parents’ educational or recognition levels and their attitudes towards respecting the child’s right to be informed about medical research and towards the necessity of explaining the treatment and research. This suggests that parents’ higher educational levels do not guarantee a higher likelihood of respecting the child’s right to decide. Gender had an impact on the necessity of explanations; mothers considered them to be more necessary than fathers did.

Furthermore, the results suggest that parents differentiate between medical care and research studies. In this study, it was clarified that Japanese parents deem it their responsibility—as someone having custody—to agree to medical research for patients who are minors; as the child’s age increases, so does the likelihood that decisions will be made through parent–child discussion and consensus-based collaborative decision-making. That said, there were fewer parents who said children themselves could make the decision than those who said parents should make the decision. This suggests that age is a significant factor in the decision-making process about child participation in research and what kind of model to adapt. This is consistent with preceding studies [27].

Therefore, the age at which children are considered to be able to make judgments concerning their own treatment and medical research participation is from 15 to 18 years old. The more serious the disease, the more the influence it has on the future; therefore, the more likely parents are to consider themselves as the final decision-makers and select the parental model. On the other hand, about half of them are oriented towards joint decision-making between parents and children. Many studies suggest collaborative decision-making enhances patient quality of life and illness management [28–31].

In this study, about 30% of parents reported wanting to be the final decision-makers in the case of life-threatening diseases, but 20% responded that they would prefer to be the decision-makers, even in the case of diseases with a chance of complete recovery. Geller reports that parents prefer to be the initial decision-makers as to whether their children will participate in research [32].

In a previous study by Varma, Jenkins, and Wendler [33], 90.5% of respondents aged 7 to 14 answered that children should participate in decision-making for research participation while only 61.5% of parents said children should be involved in the decision-making. This 2008 survey consisted of answers from 26 parents of children who participated in a study of asthma and cancer patients. The results of that study were consistent with those of the current study in that parental thinking cannot be simply compared between Japan and the United States, but in the case of serious diseases, there is a small proportion of parents who are reluctant to include their children in the decision-making process.

Since there is no general agreement or international standard with legal force in Japan and some other countries [34], it is necessary to appropriately evaluate each child’s capacity to consent in each clinical setting to involve them in the decision-making process, while simultaneously allowing them to remain subject to protection regarding decisions about complicated medical procedures. Instruments assessing children’s ability to exercise consent are currently being researched and developed (e.g., the MacCAT-CR) [35, 36]. Nonetheless, a child’s mind and body develop in tandem, and it is possible children’s personal values, along with their life experiences and personal development, differ from those of adults. It is the duty of medical professionals to inform both parents and children, and to require the involvement of both parents and children in decision-making, in order to safeguard the legal and ethical principles of informed consent.

There are some limitations to this study.

First, this Internet-based survey investigated participants’ attitudes on treatment and research in general terms. Participants’ responses might have changed in response to being asked about particular conditions, treatments, or research.

Second, the following points can be mentioned in relation to the selection bias involved in obtaining subjects using an Internet survey.

According to the results of a survey conducted by the Ministry of Health, Labor and Welfare in 2017 [37], the proportions of households who had one, two, or more than two children were 46.6, 40.3, and 13.1% respectively. In this study, the proportions of households who had one, two, and more than two children were 39.0, 45.6, and 15.5%. Regarding educational background, it was reported that in the Internet survey, people with a higher academic background were selected as the respondents, and half had a university degree or higher qualification. There is a possibility of selection bias due to the fact that a large proportion of people with many children and with more advanced educational backgrounds were recruited.

In general, Internet researchers need to consider the age of the participants because most regular Internet users tend to be younger [38]. However, given the average age of the parents of children aged 6 to 18 years old in this survey (43.9 and 40.8 years for fathers and mothers, respectively), this limitation may not have a serious effect on this particular study.

Regarding the measurement accuracy, reliability was increased by excluding contradictory answers and responses that lacked consistency in relation to the basic attributes.

## Conclusions

This study’s results suggest that although many parents favored collaborative decision-making between parents and children, they also wanted parents’ will to be reflected in the decision. However, some Japanese parents tend to believe that they should be informed before their children. Decision-making was affected by whether the condition was life-threatening, whether it could be fully treated, and whether it may result in disability, leaving open the possibility that some parents might prevent information from reaching their children. The present results also suggest that, in Japan, some parents’ opinions may become obstacles to the informed consent or assent of their children. This attitude likely results from a generally protectionist concern, due to the belief that the child does not comprehend the situation and the desire to avoid placing any undue mental or physical strain on the child. In contrast, some participants commented that informing the child might lead the child to value their treatment more strongly. In cases in which parents’ and children’s opinions differ, particularly if the child is thought to possess the capacity to consent, the extent of the child’s proper influence on the decision remains unclear. Regarding treatments and research believed to be in the child’s best interests, it is vital to assess the child’s capacity to consent, and if the child possesses that capacity their will needs to be respected. Medical professionals should aim to obtain and provide greater transparency regarding the types of information provided to parents and children, any pressure that may be applied by parents or doctors, and the parties and processes involved in decision-making. They should also work to assist parents and children in making these decisions.

## Additional file

Additional file 1: Questionnaire. (DOCX 20 kb)
