# Supplementary material for: Parents’ attitudes towards and perceptions of involving minors in medical research from the Japanese perspective
Source: BMC Med Ethics. 2018 Nov 21;19:91. doi: 10.1186/s12910-018-0330-1 (PMC6249915; doi:10.1186/s12910-018-0330-1)
Supplement: Supplementary file 1 — Questionnaire. (DOCX 20 kb) [file 12910_2018_330_MOESM1_ESM.docx]

Questionnaire

Q1 Do you have children?

1. Yes (cohabitation)
2. Yes (separation)
3. No

Q2． Please inform us of your gender.

1. male
2. female

Q3. Please indicate your age.

( ) years

Q4. Please tell us about your educational background

1 Junior high school

2 High school

3 Professional school

4 University

5 graduate school

6 Other

7 I don’t want to answer this question.

Q5

Please tell us the age of first child.

( ) years.

Q6

Please tell us the age of second child.

( )years

Q7.

Please tell us the age of third child.

( )years

Q8.

Please tell us the age of fourth child.

( )years

Q9

Please tell us the age of fifth child.

( )years

Q10.

Please tell us the annual household income

1 less than \2,000,000

2 \2,000,000 ~\2,999,999

3 \3,000,00~ \3,999,999

4 \4,000,000~ \4,999,999

5 \5,000,000~ \5,999,999

6 \6,000,000~ \6,999,999

7 \7,000,000~ \7,999,999

8 \8,000,000~ \8,999,999

9 \9,000,000 ~ \9,999,999

10 over \10,000,000

11 I don’t want to answer this question

Could you ask the question about informed consent and informed assent?

Q11 Do you know the words and contents of informed consent?

1 I Know what it is

2. I have heard about it but does not know what it entails

3 I do not know what it is.

Q12 Do you know the words and contents of informed assent?

　　　　　　　　1 I Know what it is.

2. I have heard about it but does not know what it entails.

3 I do not know what it is.

In the following questions, please select the one close to your idea　concerning medical research

Q13　Treatment method and research participation should be explained to the children

1 disagree

2 somewhat disagree

3 neutral

4 somewhat agree

5 agree

Q14 The explanation should be given to the children first before the parents 1 disagree

2 somewhat disagree

3 neutral

4 somewhat agree

5 agree

Q15 It is better not to explain the severity of the disease to the children.

1 disagree

2 somewhat disagree

3 neutral

4 somewhat agree

5 agree

Q16 The child should be informed on the risks concerning the treatment and research participation,

1 disagree

2 somewhat disagree

3 neutral

4 somewhat agree

5 agree

Q17 An explanation should be given to children according to their comprehension ability 1 disagree

2 somewhat disagree

3 neutral

4 somewhat agree

5 agree

Q18 Information guidelines how to explain concerning medical research are necessary.

1 disagree

2 somewhat disagree

3 neutral

4 somewhat agree

5 agree

Q19 If there are no other methods of treatment, it is better to enroll the child in research”

1 disagree

2 somewhat disagree

3 neutral

4 somewhat agree

5 agree

Q20 Please tell us the reasons why you would give consent to research.

Q20-1 For advanced pediatric research and future children treatment

1 disagree

2 somewhat disagree

3 neutral

4 somewhat agree

5 agree

Q20-2 I hope that participation in research will lead to the treatment of my child,

1 disagree

2 somewhat disagree

3 neutral

4 somewhat agree

5 agree

Q21 We think that there are cases where you several children or you consider children's future participation in research.

Therefore, please answer concerning the age of children assumed when making decision in research participation.

( ) years

Q22 Who decides whether or not to participate in your child's research as following cases?

Q22-1 Non-life-threatening disease/illness that can be completely cured

1. Decide by children's will
2. Parents decide
3. Follows the result of parent-child's intention agreement
4. Others

Q22-2 Non-life-threatening disease/illness but will leave a disability

1 Decide by children's will

2 Parents decide

3 Follows the result of parent-child's intention agreement

4 Others

Q22-3 Life-threatening serious disease/illness

1 Decide by children's will

2 Parents decide

3 Follows the result of parent-child's intention agreement

4 Others

Q23 　Has your child been hospitalized or hospitalized for a long time ?

1. Yes
2. No

Q24 　Have you ever been hospitalized, or have you been hospitalized for a long time?

1. Yes
2. No
